# Compreendendo a Relação entre Gordura Visceral e Saúde Cardíaca

**DOI:** 10.36660/abc.20240466

**Published:** 2025-01-22

**Authors:** Moacir Fernandes Godoy

**Affiliations:** 1 Faculdade de Medicina de São José do Rio Preto São José do Rio Preto SP Brasil Faculdade de Medicina de São José do Rio Preto (FAMERP) - Cardiologia e Cirurgia Cardiovascular, São José do Rio Preto, SP – Brasil

**Keywords:** Gordura Intra-Abdominal, Frequência Cardíaca, Sistema Nervoso Autônomo

Li com interesse o artigo “A Variabilidade da Frequência Cardíaca em Repouso está Independentemente Associada aos Escores de Classificação de Gordura Visceral em Homens Adultos Sauditas” de Syed Shahid Habib et al., publicado na ABC Cardiol^1^ comparando a variabilidade da frequência cardíaca (VFC) em 99 homens saudáveis estratificados pela classificação da gordura visceral (CGV) e mostrando associações entre a VFC e as métricas de composição corporal, mas tenho algumas reservas.

A VFC tem sido demonstrada como um método conveniente para avaliar o estado homeostático individual. Tentativas de estabelecer valores de referência de acordo com a faixa etária e grau de comprometimento têm sido numerosas na literatura especializada desde o lançamento da Task Force em 1996.^2-4^ Entre as variáveis de VFC mais estudadas estão o desvio padrão dos intervalos RR normais (SDNN) e a raiz quadrada média das diferenças sucessivas (RMSSD) no domínio do tempo e potência de baixa frequência (LF ms^2^) e potência de alta frequência (HF) além da razão LF/HF, no domínio da frequência. Essas foram exatamente as variáveis estudadas por Habib e colaboradores, concluindo que homens na categoria de menor classificação de gordura visceral (G1) apresentaram a maior VFC. A classificação de gordura visceral foi mais fortemente associada à VFC do que a porcentagem de gordura corporal e a razão massa muscular/gordura visceral (MMCGV). Os parâmetros do domínio do tempo foram mais sensíveis ao tecido adiposo visceral (TAV) do que os parâmetros do domínio da frequência. Eles concluem dizendo que os parâmetros de VFC podem ser os principais parâmetros de interesse no monitoramento do estado cardíaco-autonômico em resposta a intervenções que visam a redução do TAV. Todos esses são pontos relevantes, mas houve um problema crucial na apresentação deste estudo. Os valores de VFC citados nos resultados devem ter sofrido um erro de digitação, pois são completamente incompatíveis com os intervalos de referência.

Assim, os autores apresentam na Tabela 2 valores de SDNN de 1.776±0,229 milissegundos para o Grupo 1, 1.543±0,258 milissegundos para o Grupo 2 e 1.548±0,193 milissegundos para o Grupo 3, com valores muito semelhantes a estes para a variável RMSSD. Está bem estabelecido na literatura que tanto os valores de SDNN quanto de RMSSD raramente ultrapassam 50 milissegundos, mesmo em indivíduos saudáveis. Então, certamente houve um erro na digitação destes valores. O mesmo pode ser dito em relação à Tabela 3, que apresenta os valores das variáveis potência LF e potência HF e sua relação. Os valores de desvio padrão apresentados não são coerentes proporcionalmente com os valores médios, além disso, a relação LF/HF é extremamente baixa em alguns grupos com valores negativos, o que é impossível por se tratar de uma relação entre valores positivos.

Temo que após corrigir esses dados, a conclusão possa ser diferente, mudando a interpretação. No entanto, mesmo que não mude, devemos conhecer os valores reais das variáveis estudadas, por isso solicito respeitosamente aos autores e editores que façam as correções necessárias.
