# Supplementary material for: Compreendendo a Relação entre Gordura Visceral e Saúde Cardíaca
Source: Arq Bras Cardiol. 2025 Jan 22;122(1):e20240466. [Article in Portuguese] doi: 10.36660/abc.20240466 (PMC11805576; doi:10.36660/abc.20240466)
Supplement: Translation

# Translation: Understanding the Link between Visceral Fat and Heart Health

**DOI:** 10.36660/abc.20240466i

**Authors:** Moacir Fernandes Godoy

**Affiliations:** 1 Brazil Faculdade de Medicina de São José do Rio Preto (FAMERP) - Cardiologia e Cirurgia Cardiovascular, São José do Rio Preto, SP – Brazil

**Keywords:** Intra-Abdominal Fat, Heart Rate, Autonomic Nervous System

I read with interest the paper “Resting Heart Rate Variability is Independently Associated with Visceral Fat Rating Scores in Saudi Adult Males” by Syed Shahid Habib et al., published in ABC Cardiol^1^comparing heart rate variability (HRV) in 99 healthy men stratified by visceral fat rating (VFR) and showing associations between HRV and body composition metrics, but I have some reservations.

Heart rate variability has been demonstrated as a convenient method for assessing individual homeostatic status. Attempts to establish reference values according to age group and degree of impairment have been numerous in the specialized literature since the launch of the Task Force in 1996.^2-4^ Among the most studied HRV variables are the standard deviation of normal RR intervals (SDNN) and the Root-mean square of successive differences (RMSSD) in the time domain and Low-Frequency power (LF ms^2^) and High-Frequency power (HF) in addition to the LF/HF ratio, in the frequency domain. These were exactly the variables studied by Habib and collaborators, concluding that men in the lowest visceral fat rating category (G1) had the highest HRV. Visceral Fat Rating was more strongly associated with HRV than body fat percentage and muscle mass to visceral fat ratio (MMVFR). Time domain parameters were more sensitive to visceral adipose tissue (VAT) than frequency domain parameters. They conclude by saying that HRV parameters could be the primary parameters of interest in monitoring cardiac-autonomic status in response to interventions targeting VAT reduction. These are all relevant points but there was a crucial problem in the presentation of this study. The HRV values cited in the results must have suffered a typing error as they are completely incompatible with the reference ranges.

Thus, the authors present in Table 2 SDNN values of 1,776±0.229 milliseconds for Group 1, 1,543±0.258 milliseconds for Group 2, and 1,548±0.193 milliseconds for Group 3, with values very similar to these for the variable RMSSD. It is well established in the literature that both SDNN and RMSSD values rarely exceed 50 milliseconds, even in healthy individuals. So, there was certainly a mistake in typing these values. The same can be said in relation to Table 3, which presents the values of the variables LF power and HF power and their relationship. The standard deviation values presented are not coherent proportionally with the average values , and in addition, the LF/HF ratio is extremely low in some groups with negative values, which is impossible as it is a relationship between positive values.

I fear that after correcting these data, the conclusion may be different, changing the interpretation. Nevertheless, even if it does not change, we must know the real values of the variables studied, so I respectfully request the authors and editors to make the necessary corrections.
