# Supplementary material for: Compreendendo a Relação entre Gordura Visceral e Saúde Cardíaca
Source: Arq Bras Cardiol. 2025 Jan 22;122(1):e20240466. [Article in Portuguese] doi: 10.36660/abc.20240466 (PMC11805576; doi:10.36660/abc.20240466)
Supplement: Related texts

# Reply: Carta-resposta

**Authors:** Syed Habib, Andrew Flatt, Shaea Alkahtani

**Affiliations:** 1 King Saud University Riyadh Arábia Saudita King Saud University Riyadh - Arábia Saudita; 2 Georgia Southern University Armstrong Campus Savannah Georgia EUA Georgia Southern University - Armstrong Campus, Savannah, Georgia – EUA

Agradecemos o grande interesse do leitor em nosso artigo.^1^ Os dados de VFC são relatados em unidades transformadas em logaritmo (log10), o que não é um procedimento incomum para analisar dados de VFC não distribuídos normalmente.^2^ Embora tenhamos mencionado que os dados foram transformados em logaritmo na seção de análise estatística, lamentamos não tê-los mencionado novamente para o leitor nas descrições de tabela correspondentes, pois isso parece explicar a confusão. Além disso, os intervalos padrão variam consideravelmente, e valores acima de 50 ms são possíveis e podem até estar acima de 100 ms em alguns indivíduos saudáveis e em forma.^3,4^

## References

[1] Habib SS, Alkahtani S, Aljawini N, Habib SM, Flatt AA (2024). Resting Heart Rate Variability is Independently Associated with Visceral Fat Rating Scores in Saudi Adult Males. Arq Bras Cardiol.

[2] Romieu I, Téllez-Rojo MM, Lazo M, Manzano-Patiño A, Cortez-Lugo M, Julien P (2005). Omega-3 Fatty Acid Prevents Heart Rate Variability Reductions Associated with Particulate Matter. Am J Respir Crit Care Med.

[3] Tegegne BS, Man T, van Roon AM, Snieder H, Riese H (2020). Reference Values of Heart Rate Variability from 10-Second Resting Electrocardiograms: The Lifelines Cohort Study. Eur J Prev Cardiol.

[4] Sammito S, Böckelmann I (2016). Reference Values for Time- and Frequency-Domain Heart Rate Variability Measures. Heart Rhythm.

# Reply

**Authors:** Syed Habib, Andrew Flatt, Shaea Alkahtani

**Affiliations:** 1 Arábia Saudita King Saud University, Riyadh - Arábia Saudita; 2 USA Georgia Southern University - Armstrong Campus, Savannah, Georgia - USA

We are thankful for the reader’s keen interest in our paper.^1^ The heart rate variability data are reported in log-transformed units (log10), which is not an uncommon procedure for analyzing non-normally distributed heart rate variability data.^2^ Although we mentioned that data were log-transformed in the statistical analysis section, we regret not having mentioned it again for the reader in corresponding table descriptions, as this seems to explain the confusion. Moreover, the standard ranges vary considerably, and values above 50 ms are possible and may even be above 100 ms in some healthy, fit individuals.^3,4^
